# Postoperative elevation of CA15-3 due to pernicious anemia in a patient without evidence of breast cancer recurrence

**DOI:** 10.1186/s40792-015-0128-z

**Published:** 2015-12-30

**Authors:** Yayoi Adachi, Toyone Kikumori, Noriyuki Miyajima, Takahiro Inaishi, Eiji Onishi, Masahiro Shibata, Kenichi Nakanishi, Dai Takeuchi, Hironori Hayashi, Yasuhiro Kodera

**Affiliations:** Department of Transplantation and Endocrine Surgery, Nagoya University Graduate School of Medicine, 65 Tsurumai, Showa, Nagoya, Aichi 466-8560 Japan; Department of Gastroenterological Surgery, Nagoya University Graduate School of Medicine, 65 Tsurumaicho, Showa, Nagoya, Aichi 466-8560 Japan

**Keywords:** Breast cancer, CA15-3, Pernicious anemia

## Abstract

Cancer antigen 15-3 (CA15-3) is considered as a marker for breast cancer recurrence. However, we encountered a case where the patient showed postoperative elevation of the CA15-3 level due to pernicious anemia without evidence of breast cancer recurrence. The patient was a 60-year-old postmenopausal woman. She had undergone partial mastectomy and sentinel lymph node biopsy (SLNB) for her T1 left breast cancer. SLNB had indicated no lymph node metastases. The tumor was positive for hormone receptors and negative for human epidermal growth factor receptor 2. Therefore, an aromatase inhibitor and external beam irradiation had been administered as adjuvant therapy. However, the CA15-3 level was found to be elevated at 6 months postoperatively. Although imaging studies did not indicate breast cancer recurrence, CA15-3 levels continued to increase. Based on the findings of blood tests and gastroendoscopy, a diagnosis of pernicious anemia due to vitamin B12 deficiency was finally confirmed at 2 years and 6 months postoperatively. The CA15-3 level returned to normal after vitamin B12 administration. The possibility of pernicious anemia should be considered in cases of postoperative elevated CA15-3 levels with no evidence of recurrence in patients with early breast cancer.

## Background

Cancer antigen 15-3 (CA15-3) has been utilized as a marker for breast cancer recurrence and therapeutic effect in patients with metastatic breast cancer. However, elevated CA15-3 levels are also observed in other malignancies and nonmalignant diseases, although less frequently. Therefore, the differential diagnosis of CA15-3 level elevation in an early breast cancer patient with a relapse-free postoperative course might be difficult. We encountered a case where an early breast cancer patient had an elevated CA15-3 level due to pernicious anemia during the postoperative course.

## Case presentation

A 60-year-old postmenopausal woman visited our hospital for a routine 6-month follow-up after partial mastectomy and sentinel lymph node biopsy (SLNB) for T1 left breast cancer. She had no subjective symptoms but her CA15-3 level was found to be elevated. The patient had diabetes mellitus, and the SLNB had indicated no lymph node metastases. The histopathological findings of the resected tumor were as follows: invasive ductal carcinoma; tumor size, 10 × 8 mm; nuclear grade, 1; and surgical margin, negative. pT1N0M0, stage I cancer was diagnosed. The tumor was positive for estrogen receptor and progesterone receptor, and negative for human epidermal growth factor receptor 2 (HER2). The Ki-67 labeling index was 5 %. External beam irradiation (50 Gy/25 Fr) and letrozole had been administered as adjuvant therapy.

**Fig. 1:**
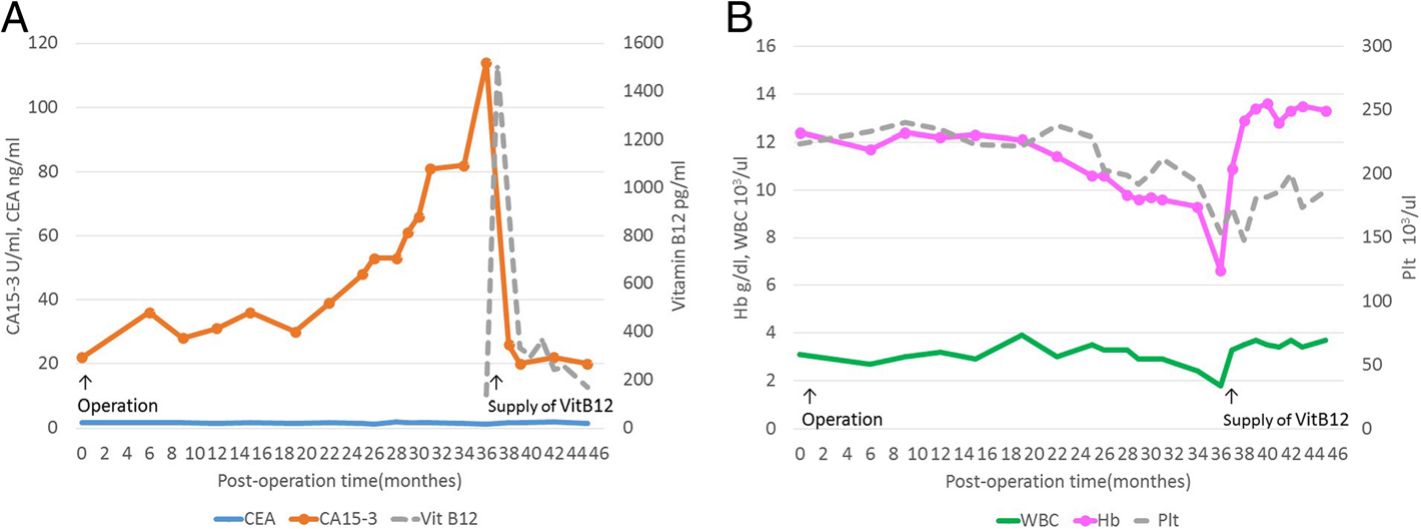
**a** Time course of the CA15-3 level. The CA15-3 level returned to normal after vitamin B12 (*Vit. B12*) administration. **b** Time course of the white blood cell (*WBC*) count, hemoglobin (*Hb*) level, and platelet (*Plt*) count. Pancytopenia improved after vitamin B12 (*Vit. B12*) administration

The CA15-3 level increased gradually to 40 U/ml 1 year postoperatively. Carcinoembryonic antigen was maintained at a normal level. Computed tomography and bone scintigraphy were performed, but there was no clinical evidence of breast cancer recurrence. At 2 years and 6 months postoperatively, the CA15-3 level increased to 80 U/ml and asymptomatic anemia was detected. Therefore, the patient was referred to the department of hematology and was hospitalized for further examinations. Positron emission tomography-computed tomography and bone marrow biopsy did not show metastatic lesions. Further blood examinations indicated pancytopenia, an elevated red blood cell mean corpuscular volume (MCV), vitamin B12 deficiency, and the presence of intrinsic factor and parietal cell antibodies. Gastroendoscopy revealed chronic atrophic gastritis. Finally, pernicious anemia was diagnosed. The CA15-3 level returned to normal after vitamin B12 administration (Fig. 1a, b). The patient is currently being followed up at an outpatient clinic without recurrence of breast cancer.

### Discussion

CA15-3 is a tumor marker used to monitor patients with certain cancers, especially breast cancer. MUC1, a cell surface glycoprotein, is released into the serum where it is referred to as CA15-3. MUC1 is expressed in both mammary epithelial cells and mammary tumor cells, although its expression on the latter is higher. Elevated serum CA15-3 levels are a common feature in metastatic breast cancer patients. CA15-3 has been recognized as a more specific marker than carcinoembryonic antigen in patients with breast cancer [1, 2]. CA15-3 level monitoring is therefore useful in postoperative surveillance of asymptomatic patients who have undergone surgery for early breast cancer and in evaluation of the therapeutic effect in metastatic breast cancer. CA15-3 elevation can detect distant metastasis in approximately 70 % of asymptomatic postoperative breast cancer patients [1]. However, an elevated CA15-3 level has been reported in normal subjects (associated with aging, pregnancy, or liver diseases) as well as in patients with other malignancies (ovarian cancer, lung cancer, hepatocellular cancer, colon cancer, prostatic cancer, pancreatic cancer, chondroid syringoma, some hematological malignancies) [3, 4]. Furthermore, there is no high-level evidence that the early detection of recurrence based on an elevation in tumor marker levels in early breast cancer patients improves their prognosis [1]. Therefore, the American Society of Clinical Oncology guideline reported that there are no data supporting the use of CA15-3 for monitoring patients for recurrence after primary breast cancer therapy [5]. However, some organizations recommend serial measurement of the CA15-3 level based on its reported usefulness for the postoperative surveillance of breast cancer patients by small studies [1]. Additionally, noninvasive and economic postoperative monitoring modalities for early breast cancer are in demand by both patients and clinical oncologists [2]. CA15-3 levels have been routinely measured at the discretion of attending physicians in our hospital.

In the case reported here, the CA15-3 level was monitored for postoperative surveillance. The patient was considered to have a low risk for recurrence in the early postoperative period based on the biological characteristics of the tumor. Initially, a nonmalignant change such as aging was considered to be the cause of CA15-3 elevation, and hence, no immediate measures were taken and the patient was followed up for 1 year. However, the CA15-3 level continued to increase gradually, and pancytopenia progressed in accordance. Therefore, carcinomatosis of the bone marrow was considered as a potential cause of pancytopenia. Accordingly, the patient was referred to the department of hematology and was ultimately diagnosed with pernicious anemia due to a vitamin B12 deficiency.

Pernicious anemia is caused by a vitamin B12 deficiency, due to chronic atrophic gastritis in the presence of autoantibodies against gastric parietal cells. Patients with solid tumors such as gastric cancer, pancreatic cancer, and gastrointestinal neuroendocrine tumors are considered to have a high possibility of pernicious anemia. However, pernicious anemia has not been reported to occur at a high incidence in patients with breast cancer [3]. Pernicious anemia can be managed with monthly intramuscular vitamin B12 injections [6].

Triantafillidis et al. first reported an elevated CA15-3 level in patients with pernicious anemia [7] in 2002. Thereafter, Symeonidis et al. reported an elevated CA15-3 level in a large number of patients with pernicious anemia [3]. However, no patients with breast cancer were included in these reports. To our knowledge, this is the first report of an elevated postoperative CA15-3 level due to pernicious anemia in a patient with a history of breast cancer and no evidence of recurrence.

MUC-1 is expressed in mammary epithelial as well as tumor cells [3]. MUC-1 is also expressed in early erythroid differentiation and is not expressed on mature erythrocytes. Pernicious anemia is associated with erythroid hyperplasia, which results in ineffective erythropoiesis. It has been hypothesized that MUC-1 is released from megaloblastic erythroblasts undergoing apoptosis in pernicious anemia patients [3]. This could explain the elevated CA15-3 level in patients with pernicious anemia.

In cases of an elevated serum CA15-3 level without apparent breast cancer recurrence, pernicious anemia due to vitamin B12 deficiency should be considered in addition to other malignancies. However, since anemia was not noted at 6 months postoperatively when the CA15-3 increase was first noted, it was difficult to determine that pernicious anemia was the cause of CA15-3 elevation at that point. The apparent causal relationship between breast cancer and pernicious anemia was not established in this case.

## Conclusions

In conclusion, the possibility of pernicious anemia should be considered in cases of elevated postoperative CA15-3 levels in patients with early breast cancer without recurrence.

## Consent

Written informed consent was obtained from the patient for publication of this case report and any accompanying images. A copy of the written consent is available for review by the Editor-in-Chief of this journal.

## Abbreviations

CA15-3: cancer antigen 15-3
HER2: human epidermal growth factor receptor 2
MCV: mean corpuscular volume
SLNB: sentinel lymph node biopsy
